# Cocaine-Induced Headache: A Review of Pathogenesis, Presentation, Diagnosis, and Management

**DOI:** 10.7759/cureus.10128

**Published:** 2020-08-30

**Authors:** Umar Farooque, Nduka Okorie, Saurabh Kataria, Syed Furqan Shah, Vijaya Chaitanya Bollampally

**Affiliations:** 1 Neurology, Dow University of Health Sciences, Karachi, PAK; 2 Neurocritical Care, AdventHealth Orlando, Orlando, USA; 3 Neurology and Neurocritical Care, University of Missouri Health Care, Columbia, USA; 4 Neurology, West Virginia University, Morgantown, USA; 5 Internal Medicine, Trinity School of Medicine, Ribishi, VCT; 6 Neurological Surgery, Capital Medical University, Beijing, CHN

**Keywords:** headache, cocaine, presentation, pathogenesis, diagnosis, management, humans, cluster headache, migraine, cocaine abuse

## Abstract

Cocaine is a vasoactive substance, and its consumption has increased throughout the world. There are many neurological complications caused by chronic cocaine use, which include headache, aneurysmal formation, ischemic stroke, hemorrhagic stroke (subdural and subarachnoid hemorrhage), seizures, etc. Headache is one of the most common symptoms that appear after cocaine use. It may occur due to dopaminergic and serotoninergic system impairment. Cocaine causes vasoconstriction by stimulation of the sympathetic nervous system and decreases the reuptake of epinephrine, norepinephrine, serotonin, and dopamine. Reversible cerebral vasoconstriction syndrome (RCVS) is well accepted with cocaine use, which occurs most commonly in middle-aged adults and females. The relation between cocaine consumption and time of occurrence of headache has been described according to which some people suffer from headaches immediately after the cocaine use, some within 40 to 90 minutes of a cocaine binge, and some even after the cocaine abstinence for long period. The diagnosis of a cocaine-induced headache depends on history, physical examination, and cerebrovascular imaging findings. And its management is done according to cause that is responsible for headache.

## Introduction and background

Cocaine use has increased across the world especially in teenagers and young adults, which contributes to lots of pathological conditions and adverse effects on health [1-3]. Brazil is one of the countries where cocaine use is very high [1]. Cocaine is the second most commonly used drug in the USA and Europe after marijuana [2]. In spite of its decreased consumption in the USA and almost stable in Europe, the worldwide cocaine consumption is increased because there is increased marketing of cocaine in countries where economic conditions are not good [1]. Many neurological complications are caused by vasoconstrictive effects and vasculitic damage of cocaine abuse, which includes headache, seizures, aneurysm formation, ischemic and hemorrhagic stroke, or subarachnoid hemorrhage [3-5].

## Review

Pathogenesis

Cocaine is an extremely vasoactive drug that has a disastrous influence on the central nervous system [5]. Headache may occur due to damage to the serotoninergic and dopaminergic system [6]. In chronic cocaine users (CCUs), a headache occurs due to synaptic depletion of dopamine and serotonin causing a condition called “empty neuron”, a classical migraine biochemical feature [7]. Cocaine causes vasoconstriction by activation of the sympathetic nervous system and decreases reuptake of catecholamine (noradrenaline and adrenaline), far ahead of other neurotransmitters (serotonin and dopamine), at presynaptic cleft and increases their supply at postsynaptic receptors and also releases epinephrine and norepinephrine by the adrenal medulla; moreover, it was investigated that serotonin and dopamine are also released by cocaine use [1,2,4,5]. In turn, dopamine activates alpha-1 receptors that cause powerful vasoconstriction. Cocaine can upsurge the number and sensitivity of synaptic dopamine receptors. Few other mechanisms are also responsible for vasoconstriction, which include activation of endothelial cells by endothelin-1 and greater calcium inflow into smooth muscle cells of vessels. Benzoylecgonine and ecgonine, the metabolites of cocaine, also have a vasoconstrictive effect and have a half-life longer than the parent drug itself. Cocaine and metabolites of cocaine have a straight deadly effect on neurons that cause greater calcium intracellularly and death of cells [5]. 

There are many pathophysiological impacts caused by cocaine consumption, which include increment in oxidative stress, raised platelet activation, raised production and activation of prostaglandins, raised sympathetic activity, and endothelial dysfunction [1,2,4]. Cocaine acts the same as class I antiarrhythmic agents and local anesthetics by blocking sodium and potassium channels [2]. Arterial thrombus formation occurs in cocaine users because of increased platelet accumulation and platelet stimulation [2,5]. Cocaine can cause tissue ischemia by several different mechanisms, such as increasing oxygen demand of tissues, constriction of vessels, and stimulation and accumulation of platelets leading to arterial thrombi [2].

The pathogenesis of cocaine-induced headaches may differ in users. In some users, the headache begins immediately after smoking cocaine or injecting it intravenously. It occurs due to a sudden surge of cocaine that rapidly blocks the presynaptic reuptake of norepinephrine and causes acute vasoconstriction. This imbalance of norepinephrine and vasomotor control seems to be an important cause of migraines. In others, a severe headache can develop during binges of cocaine use within 40 to 90 minutes after each cocaine dose. Such users may snort cocaine for temporary relief from cluster headache, which does occur due to the anesthetic effect of cocaine on sphenopalatine ganglion but the headache reappears with increasing severity because of secondary presynaptic serotonin depletion due to prolonged cocaine use. Yet another way in which headache can develop occurs in habitual users following long cocaine withdrawal. Since chronic cocaine use causes presynaptic depletion of serotonin, dopamine, and norepinephrine, the time needed for the recovery of these neurotransmitter systems may be accountable for this type of headache [8].

Previously, two cases were reported with a history of chronic cocaine use having intracerebral hemorrhage investigated on CT scan, which turned out to be biopsy-proven cerebral vasculitis when hematoma evacuation was done [9]. Reversible cerebral vasoconstriction syndrome (RCVS) is well accepted with cocaine use [10,11]. RCVS causes a headache that results from vasoconstriction due to disruption in a cerebrovascular tone, which can furthermore result in neurological complications, including stroke, if not treated early. RCVS prevalence is more in middle-aged adults and is predominant in females more importantly in the postpartum period [11]. Vascular abnormalities and aneurysms caused by cocaine use might result in subdural and subarachnoid hemorrhage [4,12]. Cocaine results in strong cerebral vasoconstriction even in healthy people, which happens more rapidly in those who had previous cocaine exposure. If CCUs stop cocaine consumption for a long period, even then perfusion deficits remain in them due to vasoconstriction [5].

Presentation

One of the most widely recognized manifestations after cocaine use is headache [6]. Headache occurs in 90% of cases of CCUs [7]. Prolonged cocaine consumption may cause headaches or deteriorate the preexisting headache with migraine or migraine-like characteristics [6,7]. Cocaine-induced headache occurs more commonly in young individuals and predominantly in females [7]. CCUs presented with cocaine-induced headaches may be divided into three groups according to the type of headache they are presented with. Group I includes those who suffer from headaches immediately after cocaine use. The headache in group I is usually presented as occipital or bilateral lasting for 2-48 hours and associated with photophobia, nausea, and vomiting. These CCUs were known to be smoking crack or injecting cocaine intravenously. Those who suffer from a headache during cocaine binges fall in group II. They used to snort cocaine or smoke it as a crack and were presented with throbbing frontal headache usually associated with nausea and sometimes vomiting. Patients suffering from a headache a few days into cocaine abstinence comprise group III. They were presented with throbbing frontal headache that progressively worsened with continued drug abstinence, associated with photophobia, nausea, and vomiting. These CCUs had a history of smoking cocaine as crack or injecting intravenously [8]. The relationship between headache and intense cocaine consumption in constant cocaine consumers has been researched in a cross-sectional investigation in a sequential arrangement. According to that study, 90% of the included individuals complained of a present headache. Previous headache history was present in 40.3% out of 90% of individuals, while in 9.7%, headache occurred after the start of cocaine consumption. Approximately 93% of those in which there was no apparent cause of headache, 35 individuals had a migraine or probable migraine without aura, 3 individuals had an episodic tension-type headache, and 2 individuals had a cocaine-induced headache. Headache worsens after cocaine use as reported by many patients [6]. A case was clinically reported with symptoms of atypical headache, cervical pain, mild abdominal pain, and hypertension, which on investigation turned out to be a dissection of vertebral arteries and right renal artery both after cocaine abuse [2]. Dissection of both extracranial carotid and vertebral arteries is not common as a result of cocaine use [10]. Few cases were reported having cluster headache criteria and substance use disorder, and cocaine was also one of those substances [13]. Luke Glancy et al. reported a case of a 49-year-old man suffering from a gradually deteriorating headache a week after smoking crack. His heart rate was 32 beats per minute and blood pressure 174/90 mm Hg. A slow heart rate and high blood pressure along with a slow respiratory rate can be an indication of increased intracranial pressure, which seems to be the responsible factor for headache [14]. Sudden severe headache after using cocaine increases suspicion about the likelihood of intracranial hemorrhage [12]. The pattern of clinical presentation and prognosis depends on the site of intracranial hemorrhage [15]. A case was reported having aneurysmal subarachnoid hemorrhage with a severe headache which was acute in onset along with nuchal rigidity [5]. 

Diagnosis

Cocaine-induced headache is diagnosed based on history, physical examination, and cerebrovascular imaging findings [11]. Brain imaging and lumbar puncture should be done in all cocaine using patients presenting with headache and in some cases angiography if needed [4]. 

Cocaine abuse decreases cerebral perfusion, which is evaluated by transcranial Doppler sonography to prevent further neurovascular complications [16]. Arteriography can be performed to determine whether multiple large vessel occlusions are present or not. Brain biopsy can be performed to detect cocaine associated with small-vessel vasculitis [17]. If intracranial hemorrhage is suspected in cocaine abuser, cerebral CT should be done to confirm if the hemorrhage is present or not; if the hemorrhage is present, then cerebral angiography reveals which artery is bleeding [12]. If a young patient clinically presents with new-onset hypertension or abdominal pain and also has a long-term history of cocaine consumption, then there are more chances that the patient may have renal artery dissection [2]. If a cocaine-related stroke is suspected based on history and examination, then the patient should undergo the proper evaluation, including toxicological confirmation of cocaine use (specimen of urine for cocaine, metabolites of cocaine and any other drug), brain imaging using magnetic resonance or CT, lumbar puncture (including Venereal Disease Research Laboratory [VDRL]), echocardiography, electrocardiogram, angiography, and hematologic studies (complete blood count [CBC], sedimentation rate, chemistry panel, fluorescent treponemal antibody [FTA] test, and coagulation studies) to exclude all other possible causes of stroke [4]. 

Management

Some CCUs use cocaine as an acute cure to treat headache outbreaks that is not effective for all cases; only 17.2% of cases benefit from it. But in more recent research, it has been notified that those patients who have incurable cluster headache that is not treated by conventional treatment, cocaine used as an acute cure shows full or partial improvement in 30.8% of cases [7]. Users suffering from headaches due to withdrawal from cocaine use can be relieved by doxepin. Doxepin may hinder transient hypoactivity of norepinephrine and serotonin during the cessation of cocaine by preventing presynaptic reuptake of these neurotransmitters [8]. Corticosteroids and cyclophosphamide treatment might prove significant to relieve cocaine users of persistent headache in some cases [17]. In cocaine abusers who reported high recurrence of migraine, amlodipine can decrease the rate of migraine and may imitate improved cerebrovascular tone [18]. The refractory cases of cocaine-induced migraine and cluster headache that are triggered by intranasal contact point stimulation can be treated by endonasal surgery [19]. Conservative treatment is recommended if there is no other cause of stroke except for cocaine abuse. Calcium channel inhibitors antagonize cocaine consequences in men [4]. Moreover, the management of cocaine-induced headache depends on the cause due to which it occurs and treated accordingly.

## Conclusions

Cocaine causes vasoconstriction by activation of the sympathetic nervous system which results in headaches. Cocaine-induced headache may also occur due to many other underlying causes such as dissection of the carotid and vertebral arteries, ischemic stroke, or hemorrhagic stroke. Therefore, any patient having headache with a history of chronic cocaine use should be taken seriously and should undergo a proper evaluation to investigate the cause of the headache and proper treatment plan should be made according to the cause of headache for a good prognosis.
